# Letter to the Editor: a commentary on ‘Impact of immediate postrecanalization cooling on outcome in acute ischemic stroke patients with a large ischemic core: prospective cohort study’

**DOI:** 10.1097/JS9.0000000000001621

**Published:** 2024-05-09

**Authors:** Lingjia Xu, Haiyan Wang

**Affiliations:** Department of Neurology, Shaoxing Second Hospital, Shaoxing, Zhejiang, People’s Republic of China

*Dear Editor,*

We reviewed a prospective cohort study by Bai *et al*.^1^ on the effect of immediate cooling after recanalization on the prognosis of patients with major ischemic stroke. Bai and his team analyzed patients following endovascular treatment for acute cerebral infarction with a large core [defined as ASPECT ≤5 on non-contrast-enhanced computed tomography (CT) or ischemic core ≥50 ml on CT perfusion]. The patients were divided into two groups: target temperature management (TTM) and non-TTM. Those in the TTM group underwent whole-body cooling targeting a core temperature of 33°C for a minimum of 48 h. The primary outcome was a good outcome at 90 days, as measured by the Modified Rankin Scale (mRS 0–2). Secondary outcomes included a good outcome at 90 days (mRS 0–3), mortality, intracranial hemorrhage within 7 days or at hospital discharge, and malignant cerebral edema. The study found that the TTM group had a higher proportion of good outcomes at 90 days compared to the non-TTM group, with no significant differences in the secondary outcomes. This suggests that cooling after vascular recanalization is feasible for patients with a large ischemic core. However, more randomized clinical trials with larger sample sizes are needed in the future to confirm its effectiveness. This study provides new treatment concepts for enhancing the success of vascular recanalization in patients with acute large-core cerebral infarctions. However, due to the limited sample size, the findings have constraints on their generalizability to clinical practice. Additionally, the study did not account for other factors that could lead to reperfusion injury, including collateral compensation and cortical venous drainage^2,3^. The authors stated that the timeframe for endovascular treatment is within 24 h, but the study was conducted between 2021 and 2022. The DEFUSE3 trial and DAWN trial ruled out large-core infarction within 16 or 24 h of anterior circulation thrombectomy^4,5^. The effect of intravenous thrombolysis was mentioned in the outcome analysis; however, whether the time window for its use in this study was 4.5 or 6 h, and whether patients presented within 4.5 or 6 h of symptom onset, the likelihood of administering intravenous thrombolysis was higher. The authors did not perform subgroup analyses to eliminate clinical heterogeneity, potentially due to a limited sample size, which necessitates a more thorough discussion to be adequately addressed. Furthermore, the patient’s weight was not considered, and when the study involves temperature control, whether the influence of the patient’s BMI and lipid index should also be considered also appears to be a potential factor.

In China, the cost of thrombectomy surgery is high. The financial situation of the patient’s family can influence the family’s choice of treatment, leading to selection bias. It is unclear whether the study accounted for the cost of the patient’s surgery. In clinical work, considering the impact of socioeconomic factors on high-cost interventional surgery can help us better choose treatment options for patients. Overall, this is a very good study and may lead to more discussion on the follow-up treatment options after recanalization in acute ischemic stroke.

## Ethical approval

Not applicable.

## Consent

Not applicable.

## Sources of funding

Not applicable.

## Author contribution

L.X.: writing; H.W.: study design.

## Conflicts of interest disclosure

The author declares no conflict of interest.

## Research registration unique identifying number (UIN)

Not applicable.

## Guarantor

Lingjia Xu and Haiyan Wang.

## Data availability statement

The data used to support the findings of this study are included within the article.

## Provenance and peer review

Not applicable.
